# Morphology-Variable Aggregates Prepared from Cholesterol-Containing Amphiphilic Glycopolymers: Their Protein Recognition/Adsorption and Drug Delivery Applications

**DOI:** 10.3390/nano8030136

**Published:** 2018-02-28

**Authors:** Zhao Wang, Ting Luo, Amin Cao, Jingjing Sun, Lin Jia, Ruilong Sheng

**Affiliations:** 1 Department of Polymer Materials, Shanghai University, 99 Shangda Road, Mailbox 152, Shanghai 200444, China; wangz@sioc.ac.cn; 2 CAS Key Laboratory of Synthetic and Self-assembly Chemistry for Organic Functional Molecules, Shanghai Institute of Organic Chemistry, Chinese Academy of Sciences, 345 Lingling Road, Shanghai 200032, China; luoting@sioc.ac.cn (T.L.); jis84@pitt.edu (J.S.); 3 CQM—Centro de Química da Madeira, Universidade da Madeira, Campus da Penteada, 9000-390 Funchal, Portugal

**Keywords:** cholesterol, galactose, morphology, lectin recognition, DOX delivery

## Abstract

In this study, a series of diblock glycopolymers, poly(6-*O*-methacryloyl-d-galactopyranose)-*b*-poly(6-cholesteryloxyhexyl methacrylate) (PMAgala-*b*-PMAChols), with cholesterol/galactose grafts were prepared through a sequential reversible addition-fragmentation chain transfer (RAFT) polymerization and deprotection process. The glycopolymers could self-assemble into aggregates with various morphologies depending on cholesterol/galactose-containing block weight ratios, as determined by transmission electronic microscopy (TEM) and dynamic laser light scattering (DLS). In addition, the lectin (Ricinus communis agglutinin II, RCA_120_) recognition and bovine serum albumin (BSA) adsorption of the PMAgala-*b*-PMAChol aggregates were evaluated. The SK-Hep-1 tumor cell inhibition properties of the PMAgala-*b*-PMAChol/doxorubicin (DOX) complex aggregates were further examined in vitro. Results indicate that the PMAgala-*b*-PMAChol aggregates with various morphologies showed different interaction/recognition features with RCA_120_ and BSA. Spherical aggregates (d ≈ 92 nm) possessed the highest RCA_120_ recognition ability and lowest BSA protein adsorption. In addition, the DOX-loaded spherical complex aggregates exhibited a better tumor cell inhibition property than those of nanofibrous complex aggregates. The morphology-variable aggregates derived from the amphiphilic glycopolymers may serve as multifunctional biomaterials with biomolecular recognition and drug delivery features.

## 1. Introduction

Synthetic glycopolymers with saccharide grafts have attracted increasing attentions due to their interesting self-assembly behavior [1] and biological functions, which guarantee their applications as advanced biomaterials such as gene/drug carriers, immunodiagnostic reagents, and bio-targeting materials [2,3,4]. The saccharide-shell-bearing aggregates/assemblies with various morphologies (e.g., nanospheres, worm-like micelles, vesicles and tubules) were obtained through the nano-precipitation [5,6] or polymerization-induced self-assembly approach [7]. Meanwhile, glyco-inside nanostructures have also been reported, and their vesicle-*to*-micelle transition could be tuned depending on the weight ratio and protection groups of the glyco-parts [8,9]. Of particular interest, the Schlaad [10] and Lecommandoux [11] groups explored glycocalyx-mimicking properties of some glycopolymeric vesicles, which showed similar physico-chemical features (such as size and structure) with natural glycocalyx architectures. For the possible applications of the glyco-containing nanostructures, molecular recognition is one of the most interested areas [12]; protein crystalline frameworks [13], protein-polymer conjugates [14], complex macroscopic self-assemblies [15], and lectin-responsive hydrogels [16] have been reported. Meanwhile, it has been revealed that the availability of this multivalent recognition could be tuned via the molecular engineering of glycopolymers [17]. Chen et al. [18] found that the block and gradient structures resulted in a superior lectin binding capability than statistical sequence copolymers, and the glycopolymer nanoparticles containing branched glycol-blocks could bind more lectins than those of their linear counterparts [19]. Zhu et al. [20] disclosed that the block glycopolymers may interact with ricinus communis agglutinin II (RCA_120_) to form larger clusters faster than the random copolymers. In addition, the surface density, neighboring functional groups, as well as morphologies of the glyco-nanoparticles could influence their lectin recognition. Nevertheless, the correlation between molecular recognition features and molecular architecture/assembly morphology for most of the glycopolymers are still obscure, which limits their biomedical applications.

On the other hand, molecular architecture/morphology of polymeric aggregates not only plays an essential role in their molecular recognition, but also in pharmacokinetics and drug delivery behaviors [21,22,23]. In particular, the aggregate morphologies (such as ellipse, cylinder, rod-like, and worm-like) have been recently disclosed to profoundly influence their endocytosis, intracellular trafficking, and tissue/organ distribution [24,25,26,27]. Mitragotri et al. [28] revealed that the particle shape instead of the size played the dominant role in phagocytosis of polystyrene particles. Likewise, poly(ethylene glycol)-block-poly(camptothecin prodrug) PEG-*b*-PCPTM self-assemblies with the morphologies of sphere, large compound vesicle (LCV), smooth disk, and staggered lamellae showed intriguing morphology-dependent cellular internalization, trafficking, and drug delivery [29]. It has also been revealed that the worm-like micelles (filomicelles) exhibited some advantages such as enhanced tumor accumulation and permeability and retention (EPR) effects [30,31], leading to prolonged blood circulation [32] and enhanced cellular uptake [33,34,35], over that of their spherical counterparts. Most recently, Gaus et al. [36] studied the cellular uptake and intracellular transport (routes) of various-shaped poly(oligoethylene glycol methacrylate)-block-poly(styrene-*co*-vinylbenzaldehyde) P(OEGMA)-*b*-P(ST-*co*-PVBA) block copolymer nanoaggregates by pair correlation microscopy, demonstrating that rod and worm-like micelles could overcome major cellular barriers better than spherical micelles and vesicles, which resulted in more doxorubicin (DOX) release inside the nucleus.

Among the amphiphilic block copolymers (BCPs), liquid crystal BCPs (LCBCPs) could spontaneously self-assemble into a number of well-ordered micro/nano objects and showed potential applications in gene/drug delivery, electronics, advanced catalysts, nanobiotechnology, etc. [37]. In particular, cholesterol as a natural rod-like mesogenic unit has already been explored for decades [38,39,40]. For instance, Zhou et al. [41] synthesized triblock copolymers with polyethylene oxide (PEO) and cholesterol attached polymethacrylates, and their self-assembled hierarchical structures were disclosed to be from lamellae to cylinder, depending on the cholesterol block contents. Likewise, disk-like self-assemblies, nanospheres, and other complex nanostructures have been reported for the cholesterol-functionalized polycarbonate copolymer amphiphiles [42,43]. In our previous studies, we prepared block copolymer amphiphiles with pendant cholesterol mesogens. They could self-assemble into micro/nanoparticles with well-ordered cholesterol mesogens in the cores of cylinder micelles, solid spheres, and bowl-shaped aggregates, and in the membranes of hollow nanotubes, ellipsoidal vesicles and so forth. [44,45,46,47,48] Additionally, as an essential component of plasma membranes, cholesterol plays important roles in cell membrane formation, adhesion, and signal transduction, regulating lipid bilayer interaction [49] and intracellular trafficking of nanoparticles [50,51]. This brings the cholesterol-based amphiphiles new potential applications in biomedical engineering [52,53,54].

To develop glycopolymer-based aggregates with tunable morphology and to further explore the effects of molecular structure/aggregate morphology on their lectin recognition and drug delivery manners, in this work, a new series of poly(6-*O*-methacryloyl-d-galactopyranose)-*b*-poly(6-cholesteryloxyhexyl methacrylate) (PMAgala-*b*-PMAChol) glycopolymers with galactose and cholesterol grafts were prepared through reversible addition-fragmentation chain transfer (RAFT) polymerization and successive trifluoroacetic acid (TFA)-mediated deprotection, and their structures were characterized. Then, self-assembled PMAgala-*b*-PMAChol aggregates with distinct morphology features were achieved by nanoprecipitation method, and the effects of glycopolymer structure/aggregate morphology on lectin binding and bovine serum albumin (BSA) adsorption were examined in aqueous media. Furthermore, employing the PMAgala-*b*-PMAChol aggregates as potential drug carriers, the DOX loading capacity, the morphology of the PMAgala-*b*-PMAChol/DOX complex aggregates, and related DOX delivery properties in human hepatocarcinoma SK-Hep-1 cells were investigated and discussed.

## 2. Experimental Section

### 2.1. Materials

6-Cholesteryloxyhexyl methacrylate (MAChol), 6-*O*-Methacryloyl-1,2:3,4-di-*O*-isopropylidene-d-galactopyranose (MAIpGP), and RAFT agent of 4-cyano-4-(dodecylsulfanyl thiocarbonyl) sulfany pentanoic acid (CDP) were synthesized and purified in similar way as described in a previous work [52]. Poly(ethylene glycol) with a molecular weight of 5000 Da (PEG-5K) and branched polyethylenimine with molecular weight of 25,000 Da (PEI-25K) were purchased from Sigma-Aldrich (St. Louis, MO, USA) and Fluka (Buchs, Switzerland), respectively. *N*,*N*′-azobis (isobutyronitrile) (AIBN, 98%, Shanghai Sinopharm Chemical Reagent Co. Ltd., Shanghai, China) was recrystallized twice in methanol prior to use. Toluene solvent was refluxed over metallic sodium, and freshly distilled before use. All other solvents and chemicals purchased from commercial suppliers were used as-received. In addition, cellulose dialysis membrane (MWCO: 3500 Da) was bought from Shanghai Green Bird Science & Technology Development Co. Ltd. (Shanghai, China). Doxorubicin (DOX, 98%) was purchased from Zhejiang Hisun Pharmaceutical Co. Ltd. (Zhejiang, China). Bovine serum albumin (BSA, Cat#0332) was supplied from Amresco (Solon, OH, USA). Thiazoyl blue tetrazolium bromide (MTT, Cat#M5655), Concanavalin A from Canavalia ensiformis (Con A), and Ricinus communis agglutinin II (RCA_120_) were all bought from Sigma-Aldrich. Human hepatocarcinoma SK-Hep-1 cells were kindly gifted by Dr. Bo Wan of the Key Laboratory of Genetic Engineering of Fudan University (Shanghai, China).

### 2.2. Analytical Procedures

^1^H NMR spectra were recorded at ambient temperature in CDCl_3_ or pyridine-*d*_5_ on a Bruker Avance-400 FT-NMR spectrometer, operated at 400.0 MHz for the proton nuclei. FTIR spectra were measured at room temperature on a Bio-Rad FTS-185 spectrometer with 64 scans, spanning a spectral range of 4000~500 cm^−1^ with a resolution of 4.0 cm^−1^. Samples were prepared by pressing dry potassium bromide (KBr) and the polymer mixture before the measurements. Oxygen elemental analysis was routinely conducted on an Elementar vario EL III system (German) in quadruplicate. Molecular weights (*M*_n_, *M*_w_) and polydispersity (*M*_w_/*M*_n_) of the synthesized polymer samples were measured at 35 °C on a PerkinElmer 200 Gel permeation chromatography (GPC) equipped with refractive index detector (RI). Tetrahydrofuran (THF) was utilized as the eluent at a flow rate of 1.0 mL/min, and a series of commercial polystyrene standards (Polymer laboratories, Stockport, UK) were employed to calibrate the GPC elution traces. Particle sizes and distribution of the prepared amphiphile self-assemblies in dilute aqueous solution were analyzed at 25 °C on a Malvern Zetasizer Nano ZS90 dynamic light scattering (DLS) instrument with incident beam at λ = 633 nm and a fixed scattering angle of 90° (Worcestershire, UK). Morphologies of the synthesized amphiphile aggregates were visualized on a transmission electronic microscope (TEM, JEOL-1230, Tokyo, Japan) under an acceleration voltage of 80 kV. In brief, the PMAgala-*b*-PMAChol aggregate aqueous solution (1.0 mg/mL) was gradually dropped onto a 300-mesh carbon-coated copper grid, and excess fluid was removed with filter paper and further air-dried under room temperature. In this study, all TEM samples were directly observed and imaged without any further staining.

### 2.3. Synthesis of Diblock PMAgala-b-PMAChol Amphiphiles

Diblock copolymer poly(6-*O*-methacryloyl-d-galactopyranose)-*b*-poly(6-cholesteryloxyhexyl methacrylate) (PMAgala-*b*-PMAChol) amphiphiles were synthesized by sequential RAFT polymerization and successive TFA-mediated deprotection. Typically, MAIpGP (1.312 g, 4.0 mmol), CDP (80.8 mg, 0.2 mmol), and AIBN (6.6 mg, 0.04 mmol) were dissolved in freshly distilled toluene (8.7 mL) and placed into a Schlenk tube equipped with a magnetic stirrer. The mixture was deoxygenated with a freeze-pump-thawing cycle a minimum of three times and then immersed into an oil bath thermostated at 80 °C for 8 h. The reaction was stopped by cooling in ice-bath, and the reaction mixture was precipitated in cold dry hexane. After filtration, the collected precipitates were dried under vacuum to finally give poly(6-*O*-Methacryloyl-1,2:3,4-di-*O*-isopropylidene-d-galactopyranose) (PMAIpGP) powders. Furthermore, the achieved PMAIpGP was employed as the macro-RAFT agent, and predetermined amounts of PMAIpGP, AIBN, and MAChol dissolved in freshly distilled toluene were in turn placed into a Schlenk tube with a magnetic stirrer. After three cycles of freeze-pump-thawing, the Schlenk tube was immersed in an oil bath preheated at 80 °C, and the reaction continued for 16 h. Then, the reaction stopped, and final products were obtained with three precipitations in anhydrous methanol and dehydration under vacuum for 12 h. Thereafter, the as-prepared PMAIpGP and PMAIpGP-*b*-PMAChols were deprotected at room temperature in mixed trifluoroacetic acid (TFA) and dichloromethane (DCM) (1/2, *v*/*v*) for 32 h, and water-soluble PMAgala and PMAgala-*b*-PMAChol block copolymer amphiphiles were achieved with good yields through repeated precipitation in anhydrous cold methanol and drying under vacuum.

PMAIpGP:

^1^H NMR (CDCl_3_, δ in ppm): 5.53 (d, Gal–H at 1 position), 4.62 (m, Gal–H at 3 position), 4.35–3.90 (m, Gal–H at 2, 4, 5 and 6 position), 2.35 (s, HOOCCH_2_R).

FTIR (in cm^−1^): 2988, 2932, 1732, 1382, 1256, 1212, 1166, 1115, 1070, 1004, 892.

PMAIpGP-*b*-PMAChol:

^1^H NMR (CDCl_3_, δ in ppm): 5.53 (Gal–H at 1 position), 5.34 (=CHR of cholesterol), 4.65 (Gal–H at 3 position), 4.35–3.75 (Gal–H at 2, 4, 5, 6 position and CH_2_COOR), 3.45 (CH_2_OR of cholesterol), 3.12 (OCHR of cholesterol).

FTIR (in cm^−1^): 2934, 2886, 1730, 1464, 1381, 1254, 1212, 1167, 1111, 1071, 1005.

PMAgala-*b*-PMAChol amphiphile:

^1^H NMR (Pyridine-*d*_5_, δ in ppm): 6.90–6.50 (–OH), 6.02–5.68 (Gal–H at 1 position), 5.55 (=CHR of cholesterol), 5.35–4.00 (Gal–H at 2, 3, 4, 5 position and CH_2_COOR), 3.65 (CH_2_OR of cholesterol), 3.38 (OCHR of cholesterol).

FTIR (in cm^−1^): 3466, 2934, 2886, 1728, 1466, 1377, 1365, 1255, 1152, 1105.

### 2.4. Self-Assembly of PMAgala-b-PMAChols in Solution

First, the critical micelle concentration (CMC) of PMAgala-*b*-PMAChols was measured by utilizing pyrene as a fluorescence probe [52]. PMAgala-*b*-PMAChol self-assemblies were conducted through nanoprecipitation in a similar way as previously published [55]. In brief, the amphiphiles were dissolved in pyridine under an initial mass concentration of 3.0 mg/mL, and deionized water was dropped slowly under gentle shaking to a water content of about 60 wt %. During this procedure, real-time mixture solution transmittance (*T*%) was measured on a UV-vis spectrophotometer (UV-2800, Hitachi, Japan) at λ = 650 nm, and the turbidities were calculated according to the equation as following:

Turbidity (%) = 2.0 − log(*T*%) (1)

When the nanoprecipitation procedure was accomplished, the mixed solution was dialyzed against deionized water using a pre-swollen cellulose membrane (molecular weight cut-off (MWCO): 3500 Da) for 48 h to remove residual organic solvent.

### 2.5. Lectin Recognition Assay

Lectin recognition of the PMAgala-*b*-PMAChol self-assemblies in aqueous solution was explored via the recording of turbidity change at λ = 450 nm vs. time at room temperature on a UV-vis spectrophotometer. Briefly, pre-determined amounts of lectin RCA_120_ were separately placed into a PMAgala_18_ solution (0.1 mg/mL) or a PMAgala-*b*-PMAChol aggregate solution (0.1 mg/mL fixed for the PMAgala block) under gentle shaking to give mixture solutions with a series of RCA_120_ mass concentration of 0.1, 0.2, 0.5, or 0.8 mg/mL, and light absorbances at λ = 450 nm were recorded per minute for 10 min with Con A and BSA as the controls.

### 2.6. BSA Adsorption Assay

Bovine serum albumin (BSA) was employed as a model to determine protein adsorption in aqueous solution for the PMAgala-*b*-PMAChol aggregates. BSA was first placed into the aggregate aqueous solution (fixed amphiphile aggregate mass concentration: 0.2 mg/mL or 0.5 mg/mL) to achieve a final BSA mass concentration of 0.5 mg/mL, and it was kept incubation at 37 °C for a predetermined period. Then, the solutions were vortexed and centrifuged at 16,000 rmp for 15 min to precipitate the BSA-adsorbed aggregates, and 1 mL supernatant of each solution was sampled. BSA mass concentration of the supernatant was evaluated at λ = 280 nm on UV-vis spectrophotometer. The amounts of BSA adsorbed on the amphiphile aggregates were thus estimated on the basis of the BSA calibration curve in a way previously reported [56]. Poly(ethylene glycol) with a molecular weight of 5000 Da (PEG-5K) and branched polyethylenimine with a molecular weight of 25,000 Da (PEI-25K) were employed as the negative and positive controls, respectively.

### 2.7. Preparation of the PMAgala-b-PMAChol/DOX Complex Aggregates

DOX-loaded complex aggregates were prepared through nanoprecipitation in a similar way as aforementioned. In brief, 27.0 mg of PMAgala-*b*-PMAChol amphiphiles and 3.0 mg of DOX were first dissolved in 10 mL pyridine, and stirred at room temperature for 10 h. Afterwards, 15 mL of deionized water was gradually dropped into the mixture under gentle agitation, and the mixture was dialyzed against deionized water for 48 h using a pre-swollen cellulose dialysis membrane (MWCO: 3500 Da) to give a DOX-loaded complex aggregate solution. DOX loading levels were further measured on a UV-vis spectrophotometer. Lyophilized DOX-loaded amphiphile aggregates were again dissolved in dimethyl sulfoxide (DMSO), and DOX mass concentration was evaluated according to a standard working curve. Thus, DOX loading content (DLC) and loading efficiency (DLE) were calculated in accordance with the following formulas [57].

(2) $$\mathrm{DLC}\left(\mathrm{wt}\;\%\right)=\frac{\mathrm{Weight}\;\mathrm{of}\;\mathrm{loaded}\;\mathrm{drug}}{\mathrm{Weight}\;\mathrm{of}\;\mathrm{loaded}\;\mathrm{drug}\;\mathrm{and}\;\mathrm{polymers}}\times 100\%$$

(3) $$\mathrm{DLE}\left(\%\right)=\frac{\mathrm{Weight}\;\mathrm{of}\;\mathrm{loaded}\;\mathrm{drug}}{\mathrm{Weight}\;\mathrm{of}\;\mathrm{drug}\;\mathrm{in}\;\mathrm{feed}}\times 100\%$$

### 2.8. Cell Viability Assay

Cytotoxicity of the as-prepared PMAgala-*b*-PMAChol aggregates was evaluated with SK-Hep-1 cells and standard method of transcriptional and translational (MTT). SK-Hep-1 cells were first seeded into a 96-well microplate (6 × 10^3^ cells/well) with Dulbecco’s Modified Eagles Medium (DMEM) supplemented with 10% fetal bovine serum (FBS), and they were incubated for 24 h. Then, the medium was aspirated and replaced with 100 μL of fresh medium containing 10% FBS, and it was supplemented with amphiphile aggregate solution under various mass concentration of 10, 30, 100, 300, and 500 μg/mL and kept cultivation at 37 °C under 5% CO_2_ for another 24 h. Afterwards, 20 μL of MTT solution (5.0 mg/mL) was placed into the microplate, the medium was replaced with 100 μL of FBS-free fresh medium and it was continuously incubated for 2 h. Then, 100 μL of DMSO was added into each well to dissolve the MTT-formazan, and light absorbances at λ = 490 nm were measured on a microplate reader (BioTek, ELX800, Winooski, VT, USA) with absorbances at λ = 630 nm as the reference. As a result, cell viability was evaluated in quintuplicate as follows:

Cell viability (%) = (OD_490 (sample)_ − OD_630 (sample)_)/(OD_490 (control)_ − OD_630 (control)_) × 100%. (4)

In a similar way, cell viability was examined for the PMAgala-*b*-PMAChol/DOX complex aggregates. After cell incubation for 24 h, the medium was aspirated and replaced with 100 μL of fresh medium (with 10% FBS), and it was supplemented with predetermined amounts of DOX-loaded complex aggregates to give a series of final DOX mass concentration of 1, 2, 3, 5, 8, 10, and 15 μg/mL. They were kept incubated at 37 °C under 5% CO_2_ for 24 h. Cell viability was accordingly evaluated as aforementioned.

## 3. Results and Discussion

### 3.1. Synthesis and Characterization of the PMAgala-b-PMAChol Amphiphilic Copolymers

**Scheme 1:**
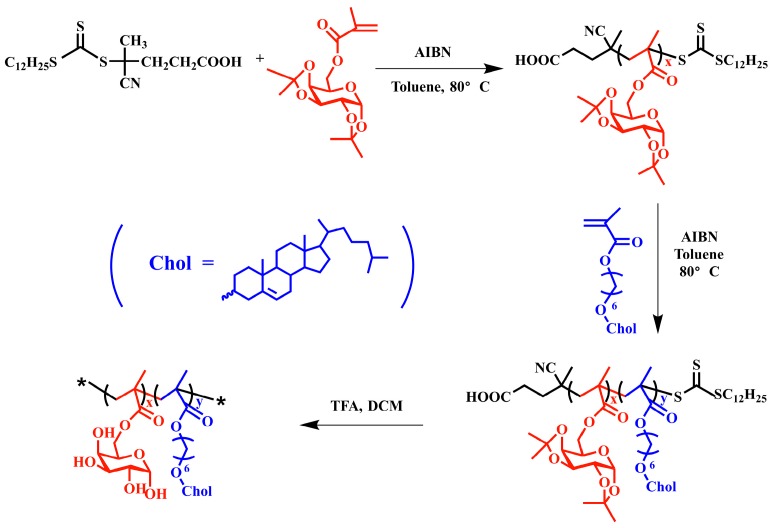
Preparation of diblock poly(6-*O*-methacryloyl-d-galactopyranose)-*b*-poly(6-cholesteryloxyhexyl methacrylate) (PMAgala-*b*-PMAChol) amphiphiles bearing galactose and cholesterol grafts.

**Figure 1:**
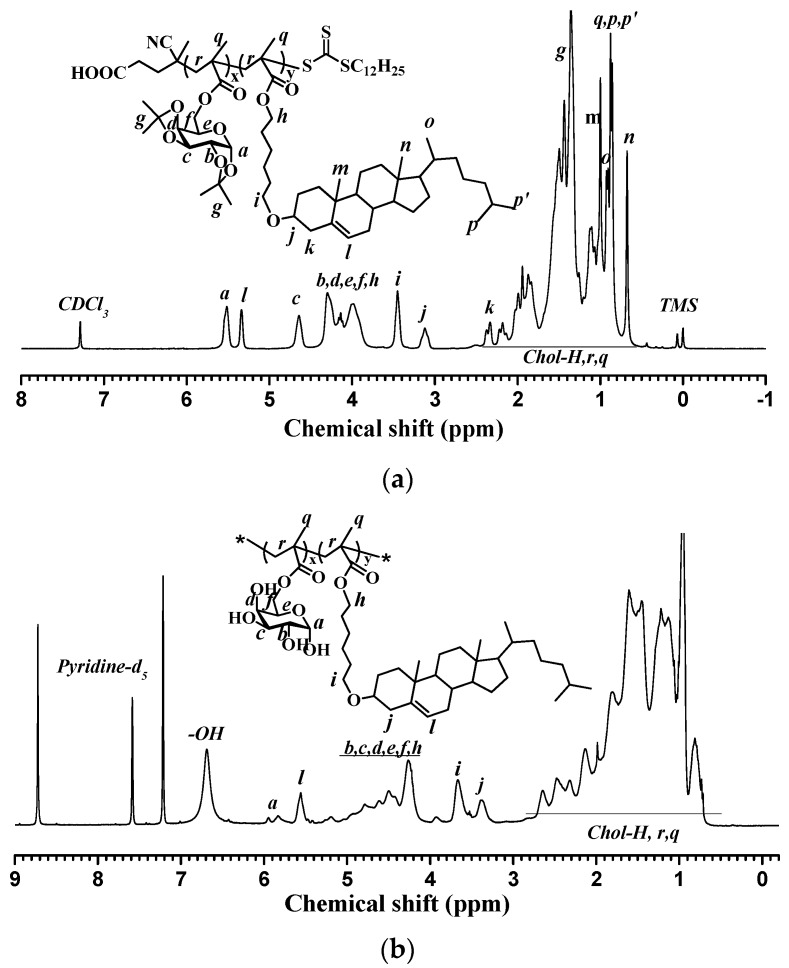

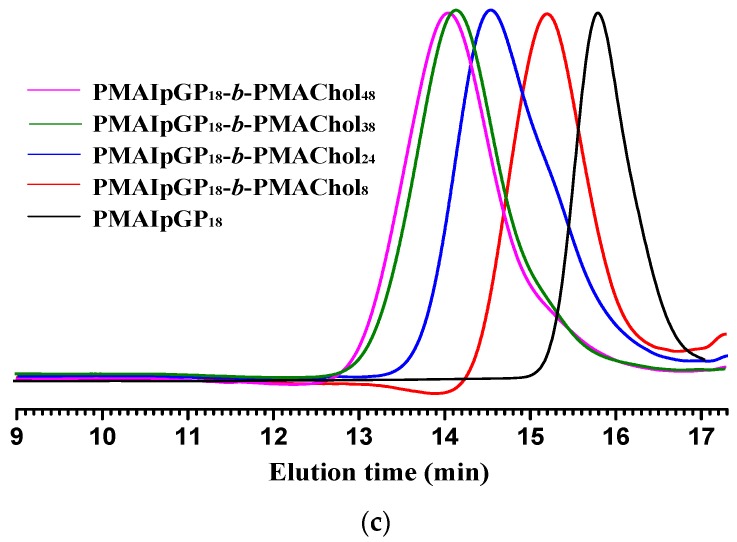
^1^H NMR spectra for the (**a**) synthesized diblock PMAIpGP-*b*-PMAChol in CDCl_3_; (**b**) PMAgala-*b*-PMAChol amphiphile in pyridine-*d*_5_ and (**c**) Gel permeation chromatography (GPC) elution traces for the PMAIpGP_18_-*b*-PMAChol series along with the PMAIpGP_18_ as macromolecular reversible addition-fragmentation chain transfer polymerization (macro-RAFT) initiator.

As illustrated in Scheme 1, a new series of diblock PMAgala-*b*-PMAChol amphiphiles were prepared through sequential RAFT polymerization and successive TFA-mediated deprotection as we recently reported [52]. Figure 1A shows a typical ^1^H NMR spectrum for the as-resulted diblock PMAIpGP-*b*-PMAChol precursor, and the proton nuclei resonance signals were accordingly assigned [41,58]. In order to achieve final PMAgala_18_-*b*-PMAChol products, a reaction condition of TFA/dichloromethane (1/2, *v*/*v*) at room temperature for 32 h was employed to deprotect the PMAIpGP_18_-*b*-PMAChol precursors, and a typical ^1^H NMR spectrum recorded in pyridine-*d*_5_ for the PMAgala_18_-*b*-PMAChol amphiphile is shown in Figure 1B, in which ^1^H nuclei resonance signals attributable to each block were observed and accordingly assigned. Furthermore, GPC traces of the PMAIpGP macro-RAFT initiator and the series of diblock PMAIpGP-*b*-PMAChol precursors are presented in Figure 1C. The monodispersive and narrow molecular weight distribution demonstrated their well-defined polymer structures. To further substantiate the diblock amphiphile structures, oxygen elemental percentages were analyzed to be 23.43 ± 0.11%, 18.95 ± 0.03%, 14.88 ± 0.09%, and 14.47 ± 0.09% for the PMAgala_18_-*b*-PMAChol_8_, PMAgala_18_-*b*-PMAChol_24_, PMAgala_18_-*b*-PMAChol_38_, and PMAgala_18_-*b*-PMAChol_48_, respectively, and these values are very close to the theoretical oxygen elemental percentages of 25.71%, 18.24%, 14.94%, and 13.92%, respectively, as estimated on the basis of their corresponding PMAIpGP_18_-*b*-PMAChol precursors. As a result, the ^1^H NMR and oxygen elemental analytical evidence could imply quantitative isopropylidene deprotection and sufficient structure stability of the diblock PMAgala_18_-*b*-PMAChols during deprotection. In this study, the synthetic results are summarized in Table 1. Notably, the synthesized PMAgala_18_-*b*-PMAChol amphiphiles have PMAChol block weight ratios of 50, 75, 83, and 86 wt %, respectively. These amphiphiles with high hydrophobic block contents were designed for self-assembling into morphology-variable “crew-cut” aggregates, which may be employed as functional nanobiomaterial models for further elucidating the effects of glycopolymer structure and aggregate morphology on biomolecular recognition/adsorption and intracellular drug delivery.

### 3.2. Self-Assembly of the PMAgala-b-PMAChol Amphiphiles in Pyridine/Water Mixed Solvent

**Figure 2:**
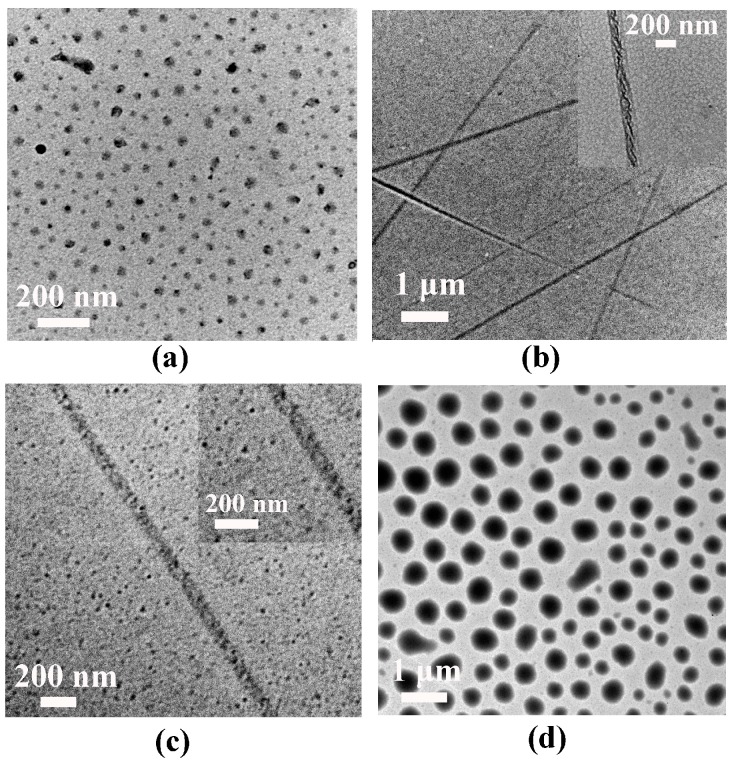
TEM morphologies of the series of amphiphile self-assemblies formed in pyridine/water mixed solution for the diblock PMAgala_18_-*b*-PMAChol_8_ (**a**); PMAgala_18_-*b*-PMAChol_24_ (**b**); PMAgala_18_-*b*-PMAChol_38_ (**c**); and PMAgala_18_-*b*-PMAChol_48_ (**d**).

To examine spontaneous aggregation, critical micelle concentration (CMC) of the PMAgala-*b*-PMAChol amphiphiles in pure water was first measured with a pyrene fluorescent probe (the results are shown in Figure S1). With an increase of PMAChol hydrophobic block length, the CMC values tend to decrease as the sequence of PMAgala_18_-*b*-PMAChol_8_ (6.82 mg/L) > PMAgala_18_-*b*-PMAChol_24_ (1.24 mg/L) > PMAgala_18_-*b*-PMAChol_38_ (0.87 mg/L) > PMAgala_18_-*b*-PMAChol_48_ (0.43 mg/L). Lower CMC values suggest their self-assembled micelles may possess relatively higher stability in water. Furthermore, self-assembly of the PMAgala_18_-*b*-PMAChol amphiphilic copolymers were implemented via gradually dropping water into their pyridine solution at ambient temperature and continuous dialyzing against deionized water for 48 h. Then, morphologies of the as-prepared aggregates under dry state were characterized by TEM. As shown in Figure 2, both PMAgala_18_-*b*-PMAChol_8_ (50/50) and PMAgala_18_-*b*-PMAChol_48_ (14/86) amphiphiles self-assembled into spherical micelles with narrow nanoparticle size distribution. Their average aggregate diameters (d) under dry state determined by TEM were ≈51 nm and 423 nm, respectively, a bit smaller than those as analyzed by DLS (≈92 nm and 543 nm) in wet state (Figure S2) due to the micelle shrinkage upon dehydration for the TEM measurements. Notably, the PMAgala_18_-*b*-PMAChol_24_ (25/75) amphiphile spontaneously self-assembled into asymmetric nanofibrous aggregates with diameter sizes of 40~200 nm and several micrometers in length. In contrast, the PMAgala_18_-*b*-PMAChol_38_ (17/83) amphiphile formed non-uniform nanoscopic spheres and fibers. To further investigate the self-assembly processes, turbidity diagrams of the mixed solution for the PMAgala_18_-*b*-PMAChol_8_ (red) and PMAgala_18_-*b*-PMAChol_24_ (blue) were measured on a UV-vis spectrometer (Figure S3). Critical aggregation water contents (CWC) of 22.5% and 13.1% were further estimated for the PMAgala_18_-*b*-PMAChol_8_ (50/50) and PMAgala_18_-*b*-PMAChol_24_ (25/75) amphiphiles, respectively. This evidence inferred that the diblock amphiphiles with higher PMAChol hydrophobic ratios tend to have higher aggregation capability via their stronger hydrophobic interactions as the driving force in the pyridine/water solution. The equilibrium turbidities of the fibrous and spherical aggregate solution were evaluated to be 0.97 and 0.05 by UV-vis for the PMAgala_18_-*b*-PMAChol_8_ (50/50) and PMAgala_18_-*b*-PMAChol_24_ (25/75), respectively. It has been reported that the colloidal turbidity could give a semi-quantitative indication of the aggregate sizes [59], therefore, the results imply that the sizes for the PMAgala_18_-*b*-PMAChol_24_ nanofibers are larger than those of the PMAgala_18_-*b*-PMAChol_8_ nanospheres.

### 3.3. Lectin Recognition of the PMAgala-b-PMAChol Aggregates

**Figure 3:**
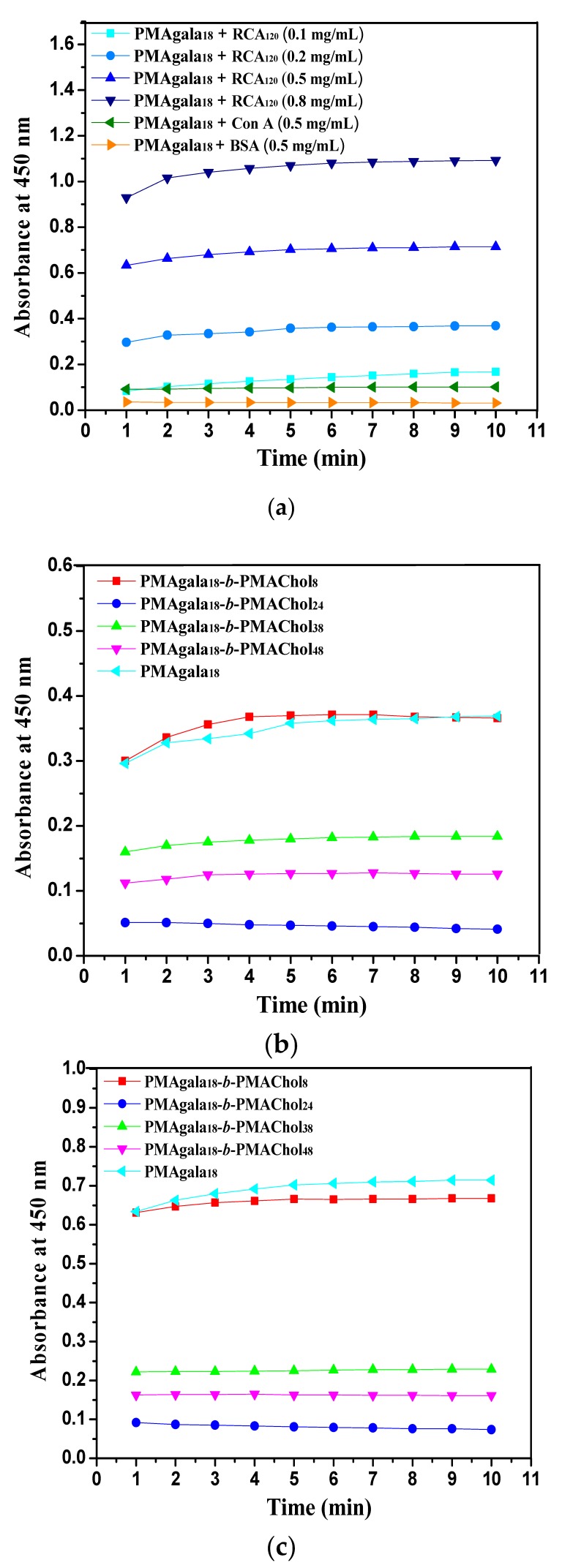
Light absorbance of aqueous solution at λ = 450 nm for the PMAgala_18_ (0.1 mg/mL) in the presence of ricinus communis agglutinin II (RCA_120_) (various concentrations), Concanavalin A from Canavalia ensiformis (Con A) (0.5 mg/mL), and bovine serum albumin (BSA) (0.5 mg/mL) (**a**) and the interactions between PMAgala_18_-*b*-PMAChol self-assemblies (0.1 mg/mL for the PMAgala block) with RCA_120_ under 0.2 mg/mL (**b**) and 0.5 mg/mL (**c**).

It is known that the specific interactions between carbohydrates and lectins play crucial roles in many biological processes like cell adhesion and hemagglutination. RCA_120_ could specifically bind galactosyl residues. Con A exhibited high affinity to the glucosyl and mannosyl residues [60], and the binding efficiencies greatly depended on the molecular structure, sugar density, and morphology of 3D glycol-nanoparticles [14,61]. To further elucidate the roles of PMAgala-*b*-PMAChol polymer structures and related aggregate morphologies played on lectin recognition, turbidimetry was employed to assay the recognition between the as-prepared PMAgala-*b*-PMAChol aggregates and lectin RCA_120_. First, homopolymer PMAgala_18_ was utilized as a model/control. As shown in Figure 3A, the PMAgala_18_ (0.1 mg/mL) strongly interacted with the RCA_120_ and the light absorbance at λ = 450 nm enhanced along with the increase of lectin concentration from 0.1 mg/mL to 0.8 mg/mL. In contrast, no significant change could be observed in the presence of Con A or BSA, demonstrating specific recognition interaction occurred between the PMAgala_18_ and lectin RCA_120_. As for the self-assembled aggregates solution, the PMAgala_18_-*b*-PMAChol_8_ spherical aggregate (d ≈ 92 nm) solution showed the highest light absorbance while the PMAgala_18_-*b*-PMAChol_24_ fibrous aggregate solution exhibited the lowest light absorbance under an RCA_120_ mass concentration of 0.2 mg/mL (Figure 3B) and 0.5 mg/mL (Figure 3C). Figure S4 depicts the pictures of the PMAgala_18_-*b*-PMAChol_8_ and PMAgala_18_-*b*-PMAChol_24_ aggregate solution before and after adding the RCA_120_. Aqueous solution with the PMAgala_18_-*b*-PMAChol_8_ spherical aggregates turned turbid with the formation of cotton-like aggregates upon the addition of lectin, furthermore, quite different particle sizes of the PMAgala_18_-*b*-PMAChol_48_ aggregates were also detected by DLS. The results suggested that the lectin RCA_120_ recognition capabilities of the PMAgala_18_-*b*-PMAChol aggregates largely relied on their 3D-morphologies. Kim et al. [62] previously reported that glycol-containing nanospheres with higher curvature (d ≈ 12 nm) have stronger binding to Con A than that of the vesicular (d ≈ 40 nm) and cylindrical objects, whilst Ladmira et al. [7] disclosed that the galactose functionalized vesicles exhibited much stronger and faster optical response upon the exposure to RCA_120_ than that of the worm-like micelles and spheres. In addition, Huang et al. [11] found that in the presence of RCA_120_, the glycopeptides with longer blocks in aqueous solution showed higher light absorbance at λ = 450 nm, nevertheless, the exact reason of the copolymer chain length and the self-assemblies structure was thereby uncertain. In this study, the results of lectin recognition in aqueous solution for the PMAgala_18_ and series of PMAgala_18_-*b*-PMAChols aggregates with the same PMAgala_18_ block ratios could undoubtedly guarantee the polymer structure and aggregate morphology, predominantly influencing the recognition between the PMAgala_18_-*b*-PMAChol aggregates and lectin; this may benefit the development of new galactose-based biomaterials for lectin detection and separation.

### 3.4. Serum Protein Adsorption of the PMAgala-b-PMAChol Aggregates

**Figure 4:**
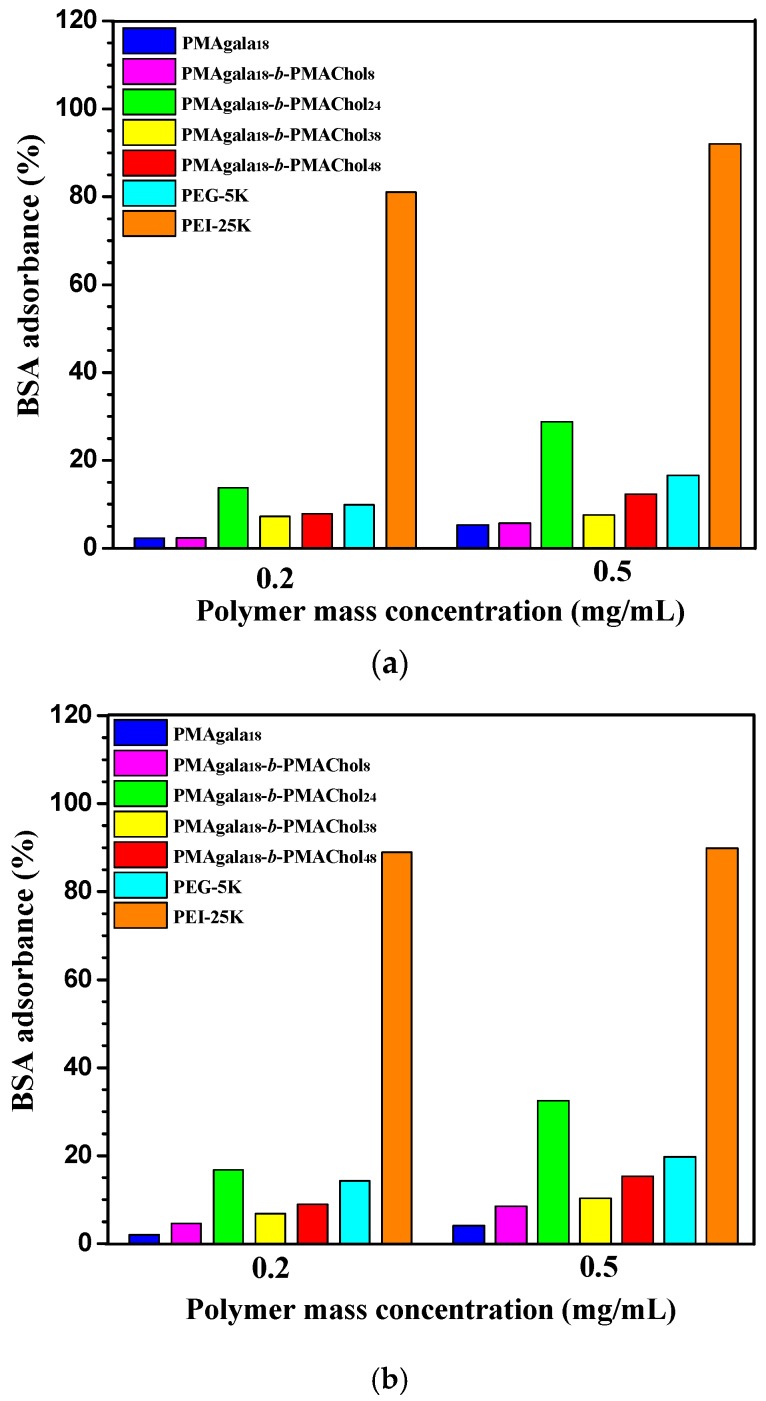
BSA adsorption assay for the PMAgala_18_ and PMAgala_18_-*b*-PMAChol aggregates under 37 °C in aqueous solution for an incubation of 3 h (**a**) and 5 h (**b**). Poly(ethylene glycol) with a molecular weight of 5000 Da (PEG-5K) and branched polyethylenimine with molecular weight of 25,000 Da (PEI-25K) were employed as the negative and positive controls, respectively. BSA protein mass concentration was preset to be 0.5 mg/mL.

So far, the adsorption of serum proteins on synthetic nanoparticle surfaces has been known to significantly affect particle internalization and pharmacokinetics, and the nanoparticle composition, size, curvature, surface potential, and hydrophobicity largely influence its protein binding profile [63]. Therefore, the design of functional nanoparticles with less serum protein adsorption seems important for realizing prolonged blood circulation in vivo. In this study, bovine serum albumin (BSA) was taken as a model protein to examine the effect of PMAgala_18_-*b*-PMAChol aggregate morphology on protein adsorption in aqueous solution. Figure 4 depicts the results of BSA adsorption assay by UV-vis. The water-soluble PMAgala_18_ and PMAgala_18_-*b*-PMAChol_8_ spherical aggregates (d ≈ 92 nm) under 0.5 mg/mL showed very low BSA adsorption, only 4.6% and 8.8% BSA were adsorbed within 5 h, much lower than that of PEG-5K negative control (19.8% BSA for 5 h). In comparison, the fibrous PMAgala_18_-*b*-PMAChol_24_ aggregates under 0.5 mg/mL gave the highest BSA adsorption, with 28.8% and 32.6% BSA adsorption after 3 h and 5 h incubation, respectively, far below that of the PEI-25K positive control (90.0% and 92.0%, respectively) due to negative surface charge of the glycol-nanoparticles [64]. Intriguingly, the different BSA adsorption for the PMAgala_18_-*b*-PMAChol aggregates may be interpreted as due to their much more distinct aggregate morphologies on lectin recognition, as discussed above. Comparing to the fibrous aggregates, the PMAgala_18_-*b*-PMAChol_8_ spherical aggregates with lower BSA binding affinities were expected to be employed as high efficient serum-resistance drug carriers for therapeutic application.

### 3.5. Intracellular Doxorubicin (DOX) Delivery by the PMAgala_18_-b-PMAChol/DOX Complex Aggregates

**Figure 5:**
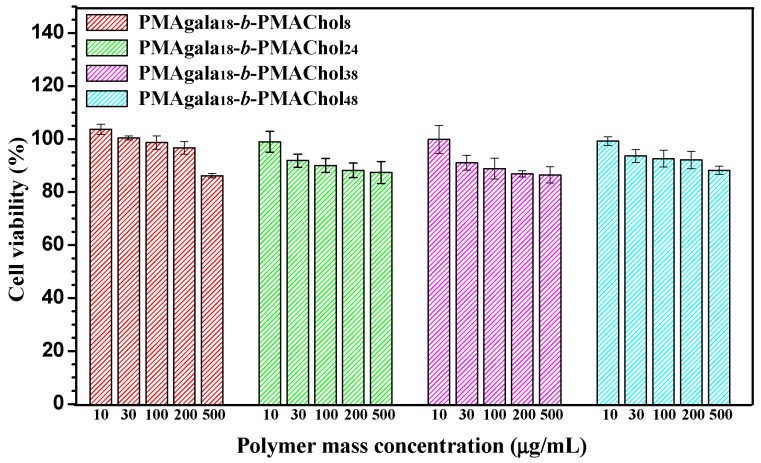
Cell viability assay for the series of PMAgala_18_-*b*-PMAChol aggregates with SK-Hep-1 cells after 24 h incubation. Data were presented as the average with standard deviation (*n* = 5).

**Figure 6:**
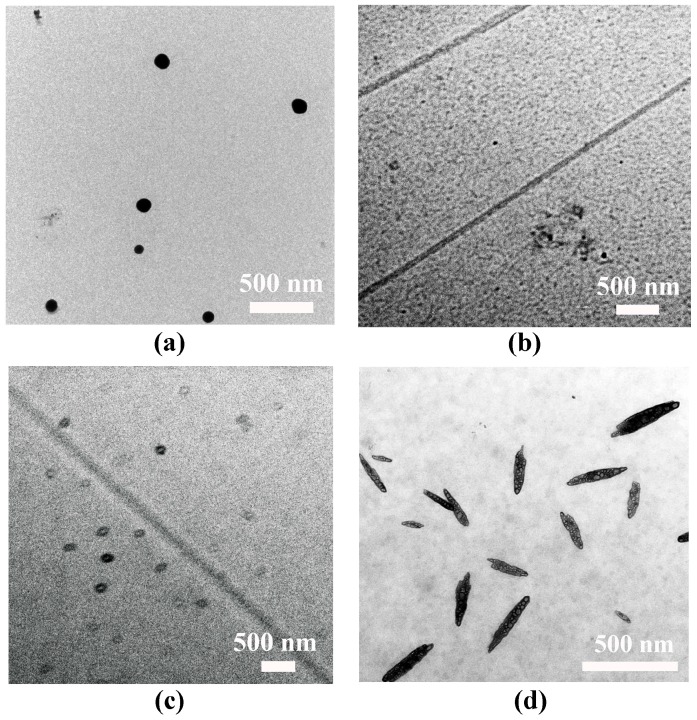
TEM morphologies for the doxorubicin (DOX)-loaded complex aggregates for the PMAgala_18_-*b*-PMAChol_8_ (**a**); PMAgala_18_-*b*-PMAChol_24_ (**b**); PMAgala_18_-*b*-PMAChol_38_ (**c**); and PMAgala_18_-*b*-PMAChol_48_ (**d**).

**Figure 7:**
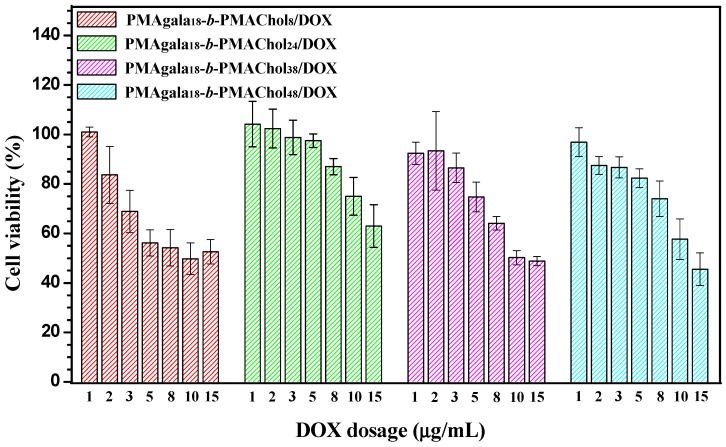
Cell viability assay for the DOX-loaded complex aggregates with SK-Hep-1 cells after 24 h incubation.

To further exploit the above-synthesized PMAgala_18_-*b*-PMAChols as drug delivery carriers, the cell toxicities were firstly evaluated by MTT assay with SK-Hep-1 cells, the results are shown in Figure 5. Under a PMAgala_18_-*b*-PMAChol concentration up to 500 μg/mL, SK-Hep-1 cell viabilities were observed higher than 90%, indicating their very low cytotoxicities in vitro. By using DOX as an anti-tumor model drug, the DOX-loaded complex aggregates PMAgala_18_-*b*-PMAChols/DOX were prepared by nanoprecipitation in pyridine/water solution, similar to the preparation condition of the above-mentioned PMAgala_18_-*b*-PMAChol aggregates. As shown in Table S1, the DLCs were measured as 8.71 wt % (PMAgala_18_-*b*-PMAChol_8_), 7.75 wt % (PMAgala_18_-*b*-PMAChol_24_), 8.26 wt % (PMAgala_18_-*b*-PMAChol_38_), and 9.33 wt % (PMAgala_18_-*b*-PMAChol_48_), and the corresponding DLEs were calculated to be 85.9%, 75.6%, 81.0%, and 92.6%, respectively. Morphologies of the PMAgala_18_-*b*-PMAChol/DOX complex aggregates were investigated by TEM (Figure 6), and the self-assembled aggregates with various aggregate morphologies including nanospheres (PMAgala_18_-*b*-PMAChol_8_/DOX), nanofibers (PMAgala_18_-*b*-PMAChol_24_/DOX), mixture of nanospheres/nanofibers (PMAgala_18_-*b*-PMAChol_38_/DOX), and nanospindles (PMAgala_18_-*b*- PMAChol_48_/DOX) were observed, indicating the DOX-loaded morphology-variable aggregates could be obtained by using the certain amphiphilic glycopolymer as the drug carrier. To study the DOX delivery efficiency of the as-prepared morphology-variable aggregates, SK-Hep-1 cell viabilities were examined by MTT assay. It could be seen that the tumor cell proliferation inhibition strongly depended on DOX dosage, and cell viabilities decreased to 45~63% under the DOX dosage of 15 μg/mL (Figure 7), inferring efficient endocytosis and intracellular DOX release of the DOX-loaded complex aggregates. Noteworthy, the SK-Hep-1 cell proliferation greatly depended on the morphologies of the complex aggregates, the spherical complex aggregates (PMAgala_18_-*b*-PMAChol_8_/DOX) showed the highest cell proliferation inhibition efficiency, whilst the nanofibrous aggregates (PMAgala_18_-*b*-PMAChol_24_/DOX) exhibited the lowest cell inhibition under the same DOX dosage. Moreover, the IC_50_ (half maximal inhibitory concentration) values were evaluated to be 9.06, 26.70, 13.54, and 14.36 μg DOX equiv./mL for the above complex aggregates, respectively. The results may due to their different molecular structure/morphology-dependent cellular interactions and drug release; the spherical complex aggregates have comparably smaller size and larger surface area than the aggregates with other morphologies, which may endow them with their higher cellular uptake and drug release manners. Likewise, Zheng et al. [65] reported that drug release behavior was determined by the matrix morphologies and the interactions between drug and matrix, and the methoxy poly(ethyleneglycol)-poly(lactic acid) (mPEG-PLA) nanofibrous vectors showed BSA release slower than that of their nanoparticle counterparts with corresponding kinetic *t*_1/2_ (time for 50% drug release) of 175.5 h and 11.76 h, respectively. Alternatively, methotrexate (MTX) decorated MPEG-PLA nanobacillus (MPEG-PLA-MTX NB) prepared by Hou et al [35] were shown to enhance cell internalization, accumulation, and tumor inhibition superiorly to that of the MPEG-PLA-MTX spherical nanoparticles in vivo. In addition, the IC_50_ values of a series of poly[oligo(ethyleneglycol) methacrylate]-block-[poly(styrene)-*co*-poly(vinyl benzaldehyde)] (POEGMA-*b*-P(ST-*co*-VBA)-DOX) nanoparticles with distinct morphologies were found to decrease in an order from micelles, vesicles, rods to worm-like nanoparticles with MCF-7 breast cancer cells [34]. In fact, until now, there was a lack of in-depth studies for revealing the morphological effects of nanoscale drug carriers on their interaction with cells and subsequent intracellular trafficking and drug release. In this work, the preliminary study on PMAgala_18_-*b*-PMAChols/DOX complex aggregates as drug carriers demonstrated an obvious molecular structure/morphology-dependent intracellular DOX release and tumor cell proliferation inhibition, which may provide potential platforms for designing highly efficient drug delivery nanosystems.

## 4. Conclusions

In summary, a new series of diblock PMAgala_18_-*b*-PMAChol amphiphilic glycopolymers with galactose and cholesterol grafts were designed and prepared. These glycopolymers could self-assemble into morphology-variable nanoscale aggregates from spherical nanoparticles to nanofibers. These glycol-containing amphiphilic aggregates showed different lectin RCA_120_ recognition and BSA adsorption behaviors, largely relying on their molecular structure/aggregate morphology. MTT assay of the PMAgala_18_-*b*-PMAChol aggregates (up to 500 μg/mL) indicated their low cytotoxicity toward SK-Hep-1 cells, enabling them to serve as promising drug carriers in vivo. Furthermore, the PMAgala_18_-*b*-PMAChol/DOX complex aggregates were prepared, and the DOX-loading and cell proliferation inhibition properties of the complex aggregates were also found to be molecular structure/morphology-dependent. The spherical complex aggregates gave rise to higher tumor cell inhibition efficiency than those of the nanofibrous and nanospindles counterparts under the same DOX dosages. The study may provide a potential molecular structure/morphology-control approach towards the design and preparation of efficient amphiphilic glycopolymers for protein recognition/adsorption and drug delivery.

## Figures and Tables

**Table 1:** Synthetic results for the PMAIpGP macro-RAFT initiator and diblock PMAIpGP-*b*-PMAChols.

Entry ^1^	Sample	Monomer Conversion (%) *^2^*	Molecular Weight				PMAgala/PMAChol Ratio (wt %)
			*M*_n, thero_^3^ (kg mol^−1^)	*M*_w, GPC_^4^ (kg mol^−1^)	*M*_n, GPC_^4^ (kg mol^−1^)	*M*_w_/*M*_n_^4^	
1	PMAIpGP_18_	91	6.37	5.56	4.95	1.10	-
2	PMAIpGP_18_-*b*-PMAChol_8_	82	10.94	12.56	10.95	1.14	50/50
3	PMAIpGP_18_-*b*-PMAChol_24_	96	19.74	20.84	16.23	1.28	25/75
4	PMAIpGP_18_-*b*-PMAChol_38_	96	27.76	19.83	24.74	1.23	17/83
5	PMAIpGP_18_-*b*-PMAChol_48_	95	32.83	36.19	31.23	1.15	14/86

Notes: ^1^ Initial 4-cyano-4-(dodecylsulfanyl thiocarbonyl) sulfany pentanoic acid/6-*O*-Methacryloyl-1,2:3,4-di-*O*-isopropylidene-d-galactopyranose/*N*,*N*′-azobis (isobutyronitrile) (CDP/MAIpGP/AIBN) feeding molar ratio of entry 1 was preset to be 1.0/20.0/0.2, and the PMAIpGP/6-Cholesteryloxyhexyl methacrylate (MAChol)/AIBN feeding molar ratio of entry 2~5 were set to be 1.0/10.0/0.2, 1.0/25.0/0.2, 1.0/40.0/0.2 and 1.0/50.0/0.2, respectively. ^2^ Monomer conversions were estimated by ^1^H NMR in CDCl_3_. ^3^ Theoretical number average molecular weights (*M*_n_) were calculated on the basis of corresponding monomer conversion. ^4^ Data express the molecular weights and distribution determined by GPC with polystyrene standard calibration.

## Supplementary Materials

The following are available online at http://www.mdpi.com/2079-4991/8/3/136/s1. Figure S1: Fluorescence intensity ratios (I394/I374) as a function of logarithm of PMAgala_18_-*b*-PMAChol mass concentration in water. Figure S2: Particle sizes and distributions for the amphiphile self-assemblies formed by diblock PMAgala_18_-*b*-PMAChol_8_ and PMAgala_18_-*b*-PMAChol_48_ by DLS (A,C) and TEM (B,D), respectively. Figure S3: Turbidity profiles for the PMAgala_18_-*b*-PMAChol_8_ (A) and PMAgala_18_-*b*-PMAChol_24_ (B) in pyridine/water mixed solution with initial mass concentration of 3.0 mg/mL in pyridine, and the inset demonstrated the photograph of amphiphile aggregate solutions for the PMAgala_18_-*b*-PMAChol_8_ (A) and PMAgala_18_-*b*-PMAChol_24_ (B). Figure S4: Photographs of the PMAgala_18_-*b*-PMAChol_8_ (A,B) and PMAgala_18_-*b*-PMAChol_24_ (C,D) aggregate solutions before and after adding RCA_120_. Particle sizes and distributions were analyzed by DLS for the PMAgala_18_-*b*-PMAChol_8_ self-assemblies in the absence (blue curve) and the presence (red curve) of RCA_120_ (E). Table S1: Characteristics of the doxorubicin (DOX)-loaded complex nanoparticles by diblock PMAgala_18_-*b*-PMAChol amphiphiles.
