# Supplementary material for: Morphology-Variable Aggregates Prepared from Cholesterol-Containing Amphiphilic Glycopolymers: Their Protein Recognition/Adsorption and Drug Delivery Applications
Source: Nanomaterials (Basel). 2018 Feb 28;8(3):136. doi: 10.3390/nano8030136 (PMC5869627; doi:10.3390/nano8030136)
Supplement: Supplementary file 1 [file nanomaterials-08-00136-s001.pdf]

Supplementary Information

Morphology-Variable Aggregates Prepared from Cholesterol-Containing Amphiphilic Glycopolymers: Their Protein Recognition/Adsorption and Drug Delivery Applications

Zhao Wang [1,2], Ting Luo [2], Amin Cao [2,*], Jingjing Sun [2], Lin Jia [1,2,*] and Ruilong Sheng [2,3,*]

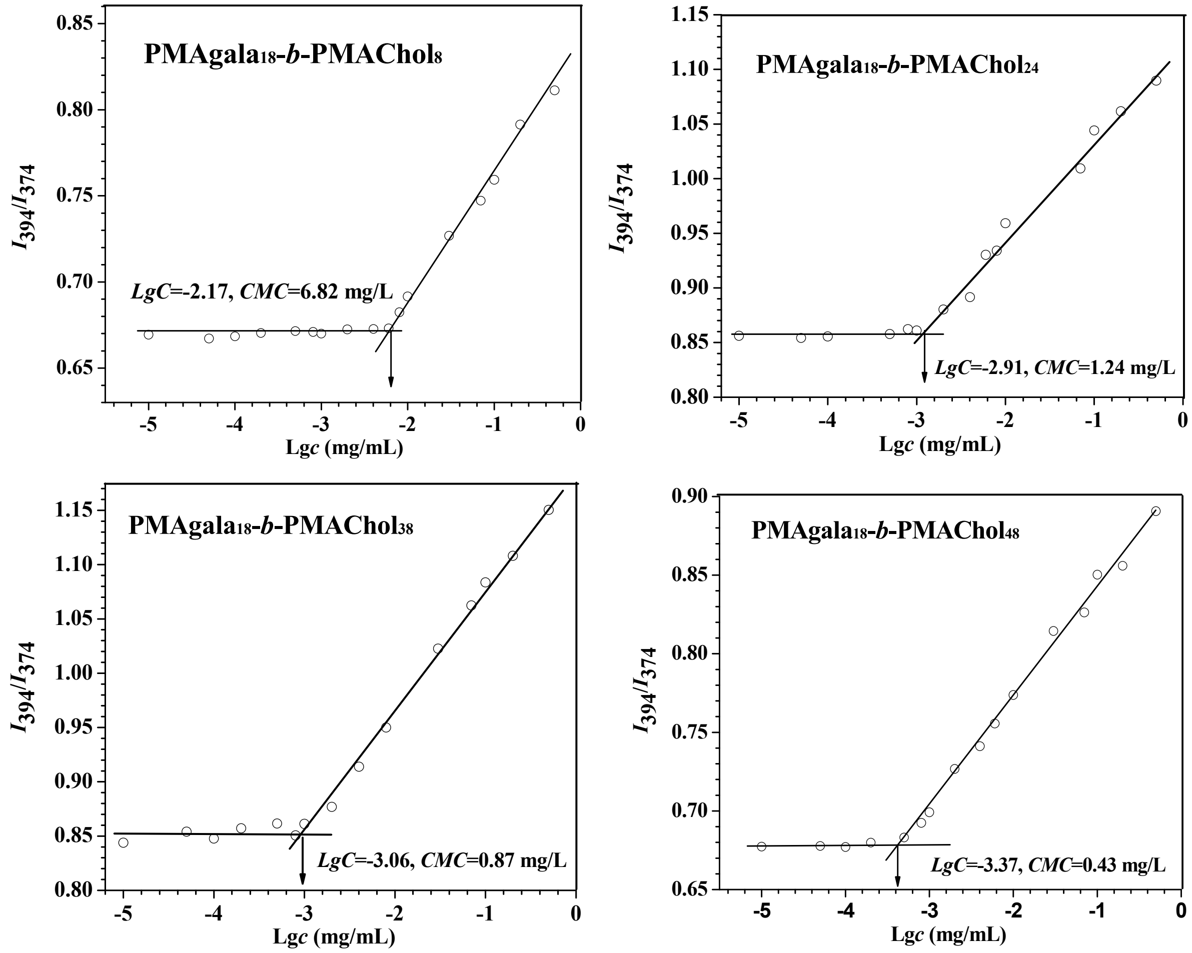

Figure S1. Fluorescence intensity ratios (I_{394}/I_{374}) as a function of logarithm of PMAgala$_{18}$-*b*-PMAChol mass concentration in water.

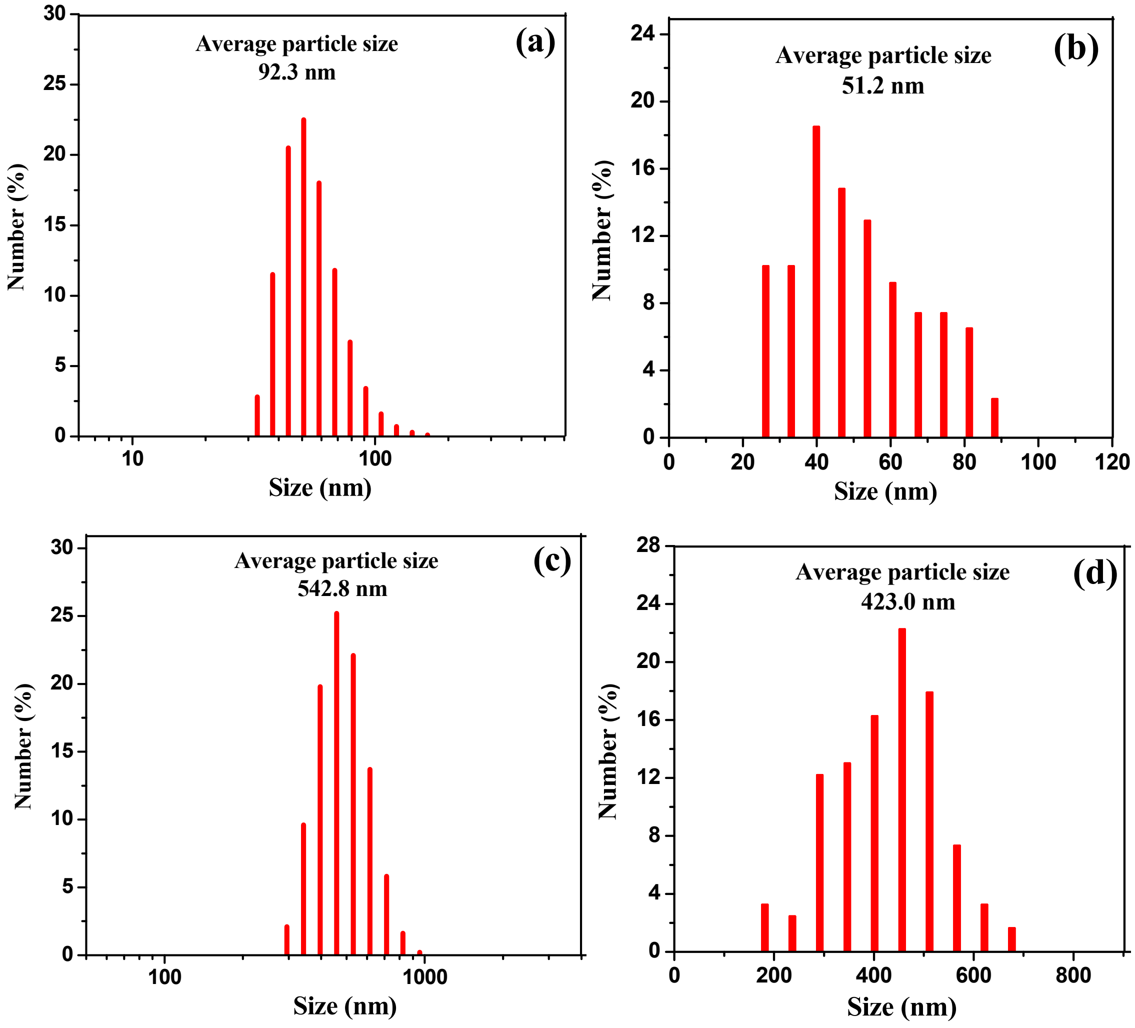

Figure S2. Particle sizes and distributions for the amphiphile self-assemblies formed by diblock PMAgala$_{18}$-*b*-PMAChol$_{8}$ and PMAgala$_{18}$-*b*-PMAChol$_{48}$ by DLS (**a**,**c**) and TEM (**b**,**d**), respectively.

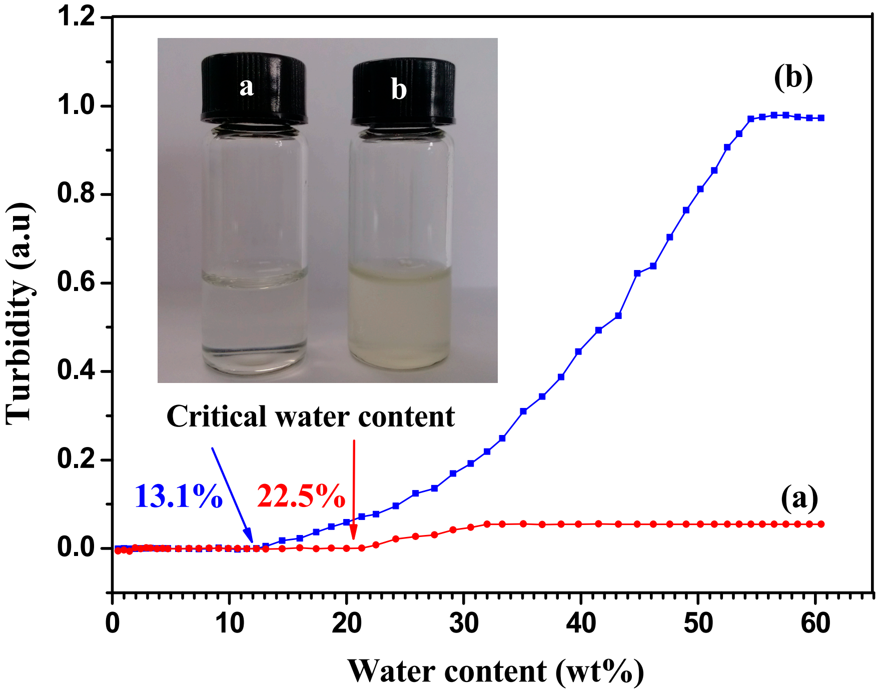

Figure S3. Turbidity profiles for the PMAgala$_{18}$-*b*-PMAChol$_{8}$ **(a)** and PMAgala$_{18}$-*b*-PMAChol$_{24}$ **(b)** in pyridine/water mixed solution with initial mass concentration of 3.0 mg/mL in pyridine, and the inset demonstrated the photograph of amphiphile aggregate solutions for the PMAgala$_{18}$-*b*-PMAChol$_{8}$ **(a)** and PMAgala$_{18}$-*b*-PMAChol$_{24}$ **(b)**.

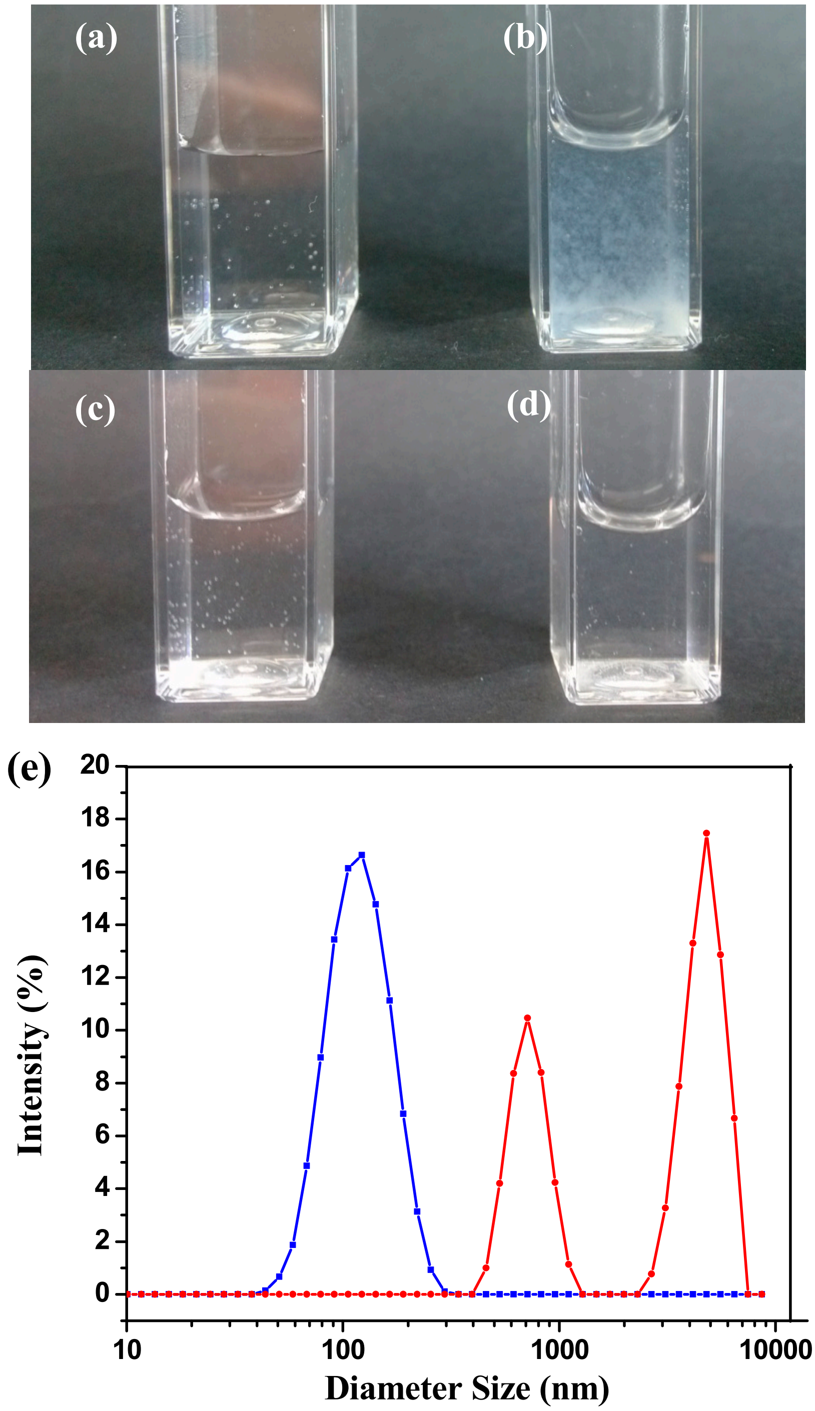

Figure S4. Images of the self-assembled aggregate solutions before and after adding RCA_{120} for the $PMAgala_{18}$-*b*-$PMAChol_{8}$ (**a**,**b**) and $PMAgala_{18}$-*b*-$PMAChol_{24}$ (**c**,**d**), respectively. Particle sizes and distributions were analyzed by DLS for the $PMAgala_{18}$-*b*-$PMAChol_{8}$ self-assemblies in the absence (blue curve) and the presence (red curve) of RCA_{120} **(e)**.

Table S1 Characteristics of the Doxorubicin (DOX)-loaded complex nanoparticles by diblock PMAgala$_{18}$-*b*-PMAChol amphiphiles.

Formulations	DLC (wt %) [1]	DLE (%) [1]	Nanoparticle Morphologies [2]	IC_{50} (μg DOX equiv./mL)[3]
PMAgala$_{18}$-*b*-PMAChol$_{8}$/DOX	8.71	85.9	spheres	9.05
PMAgala$_{18}$-*b*-PMAChol$_{24}$/DOX	7.75	75.6	fibers	26.70
PMAgala$_{18}$-*b*-PMAChol$_{38}$/DOX	8.26	81.0	spheres+ fibers	13.54
PMAgala$_{18}$-*b*-PMAChol$_{48}$/DOX	9.33	92.6	spindles	14.36

Notes: [1] Data were calculated with a theoretical DOX loading content of 10.0 wt %. [2] Complex nanoparticle morphologies were visualized by TEM. [3]IC_{50} values were assayed after 24 h incubation with SK-Hep-1 cells
